# The additive effect of herbal medicines on lifestyle modification in the treatment of non-alcoholic fatty liver disease: a systematic review and meta-analysis

**DOI:** 10.3389/fphar.2024.1362391

**Published:** 2024-02-23

**Authors:** Myung-Ho Kim, Subin Ahn, Nayeon Hur, Seung-Yun Oh, Chang-Gue Son

**Affiliations:** ^1^ Liver and Immunology Research Center, Daejeon Korean Medicine Hospital of Daejeon University, Daejeon, Republic of Korea; ^2^ Department of Internal Korean Medicine, Woosuk University Medical Center, Jeonju, Republic of Korea; ^3^ Department of Sasang Constitutional Medicine, Woosuk University Medical Center, Jeonju, Republic of Korea

**Keywords:** non-alcoholic fatty liver disease, herbal medicine, lifestyle modification, systematic review, meta-analysis

## Abstract

**Introduction:** Non-alcoholic fatty liver disease (NAFLD) is difficult to manage because of its complex pathophysiological mechanism. There is still no effective treatment other than lifestyle modification (LM) such as dietary modifications, regular physical activity, and gradual weight loss. Herbal medicines from traditional Chinese Medicine and Korean Medicine have been shown to be effective in the treatment of NAFLD based on many randomized controlled trials. This systematic review and meta-analysis aims to evaluate the additive effects of herbal medicines on LM in the treatment of NAFLD.

**Methods:** Two databases (PubMed and Cochrane library) were searched using keywords related to NAFLD and herbal medicines. Then the randomized controlled trials (RCTs) evaluating the therapeutic effects of herbal medicines combined with LM were selected. The pooled results were analyzed as mean difference (MD) with 95% confidence interval (CI) for continuous data, and risk ratio (RR) with 95% CI for dichotomous data.

**Results and Discussion:** Eight RCTs with a total of 603 participants were included for this review study. Participants were administered with multi-herbal formulas (Yiqi Sanju Formula, Tiaogan Lipi Recipe, and Lingguizhugan Decoction) or single-herbal extracts (Glycyrrhiza glabra L., Magnoliae offcinalis, Trigonella Foenum-graecum L. semen, Portulaca oleracea L., and Rhus Coriaria L. fructus) along with LM for 12 weeks. The meta-analysis showed a significant improvement in ultrasoundbased liver steatosis measured by odds ratio (OR) in the herbal medicine group than those with LM alone (OR = 7.9, 95% CI 0.7 to 95.2, *p* < 0.1). In addition, herbal medicines decreased the levels of aspartate transferase (MD -7.5, 95% CI -13.4 to −1.7, *p* = 0.01) and total cholesterol (MD -16.0, 95% CI -32.7 to 0.7, *p* = 0.06) more than LM alone. The meta-analysis partially showed clinical evidence supporting the additive benefits of herbal medicines for NAFLD in combination with LM. Whereas, it is necessary to provide a solid basis through higher-quality studies using a specific herbal medicine.

## 1 Introduction

Non-alcoholic fatty liver disease (NAFLD) is a condition in which excess fat (>5–10% of the liver weight) accumulates in the liver without excessive drinking, resulting in steatohepatitis, liver fibrosis, and cirrhosis (Younossi et al., 2021). With a prevalence of 30%–40% worldwide, NAFLD has become a widespread health concern (Im et al., 2021; Riazi et al., 2022). NAFLD has the potential to progress to cirrhosis, hepatocellular carcinoma (HCC), and death. The escalating prevalence of NAFLD in the general population highlights the increasing role of NAFLD in HCC epidemiology (Fernando et al., 2019). NAFLD sometimes develops into HCC without progressing to liver fibrosis or cirrhosis, therefore early diagnosis and management of liver steatosis are important (Berkan-Kawińska and Piekarska, 2020).

However, there is still no effective treatment other than lifestyle modification (LM) for NAFLD (Younossi et al., 2021). While there has been progress in understanding how NAFLD develops and finding potential treatments, significant challenges persist. There is currently no medication specifically approved for NAFLD (Friedman et al., 2018). The pathophysiological mechanisms of NAFLD are complex and involve systemic metabolic dysfunction and inflammation, making it difficult to manage NAFLD with single-target drugs. Therefore, combination therapies modulating multiple targets have recently been investigated (Makri et al., 2022). In traditional Chinese Medicine and Korean Medicine, herbal medicines are multi-compound and multi-target drugs that appear to have potential for the prevention and treatment of NAFLD (Dai et al., 2021). However, its effectiveness has not yet been clearly demonstrated.

Many randomized controlled trials (RCTs) have been conducted on herbal medicines for the treatment of NAFLD over 2 decades, which suggest that herbal medicines improve NAFLD and are superior to conventional drugs such as silymarin and ursodeoxycholic acid (Lee et al., 2022). As mentioned earlier, LM should not be overlooked when considering any treatment modalities.

Therefore, the purpose of this study was to find out whether herbal medicines have additive effect on LM in the treatment of NAFLD.

## 2 Methods

### 2.1 Search strategy

This systematic review and meta-analysis was conducted based on the PRISMA guidelines. Two major databases (PubMed and Cochrane library) were searched using keywords related to NAFLD and herbal medicine through February 2023.

### 2.2 Selection criteria

The studies that met the following criteria were included: RCTs evaluating the therapeutic effects of herbal medicines which are used in traditional Chinese Medicine and traditional Korean Medicine, combined with LM for NAFLD. The studies that did not use placebo as a control were excluded. There was no limit on the language.

### 2.3 Risk of bias assessment

The quality of the included RCTs was evaluated using the Cochrane library risk of bias assessment tool. The 7 items used to assess bias in each trial included random sequence generation, allocation concealment, double blindness of participants and trial performers, blindness of outcome assessment, incomplete outcome data, selective reporting, and other biases. Each quality item was divided and categorized into high risk, low risk, and unclear. This work was completed by two independent reviewers, and a third was responsible for resolving controversial issues.

### 2.4 Data extraction and review process

After screening the title and abstract of all the studies, the full text of the relevant articles was assessed by two reviewers. We conducted a systematic review on the additive effect of herbal medicines on LM in the treatment of NAFLD. We extracted the following data: name of the authors, patient information, sample size, name of herbal medicine, duration of herbal medicine treatment, observation period, and outcome measurements [ultrasound (US) liver steatosis grade, computed tomography (CT) liver/spleen ratio, body mass index (BMI), homeostatic model assessment for insulin resistance (HOMA-IR), alanine transaminase (ALT), aspartate transaminase (AST), gamma-glutamyl transferase (GGT), triglyceride, and total cholesterol] in the study.

A meta-analysis was performed using odds ratio (OR) for the improvement of US liver steatosis grade, and mean difference (MD) for the CT liver/spleen ratio, BMI, HOMA-IR, ALT, AST, GGT, triglyceride, and total cholesterol with 95% confidence interval (CI). Random-effect models were used due to heterogeneity. Dichotomous data are expressed as the OR with 95% CI. MD with the 95% CI were calculated for continuous data. Statistical significance was set at *p* < 0.05. Review Manager 5.4.1 was used for the analysis (http://www.tech.cochrane.org/revman) (accessed on 13 January 2023).

## 3 Results

### 3.1 Characteristics of the included studies

**FIGURE 1:**
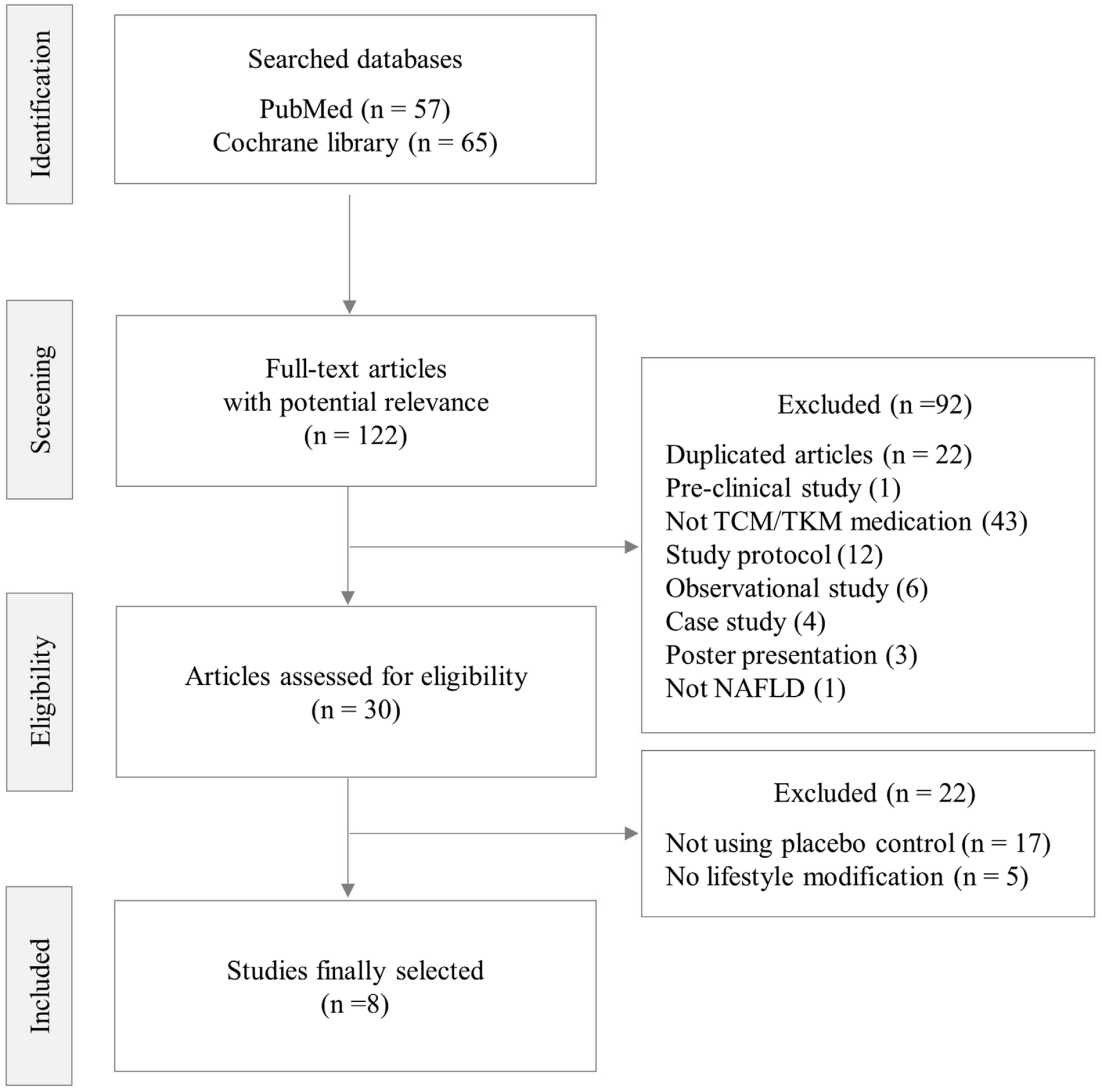
Flowchart of the literature search and study selection.

From 249 articles initially searched, 8 studies finally met the criteria of this review, which enrolled 603 participants (male 302, female 301, 326 in LM plus herbal medicine versus 277 in LM plus placebo) (Figure 1; Table 1). Risk of bias of each included study was generally assessed as low (Supplementary Figures S1A, S1B).

**TABLE 1:** Characteristics of the included 8 studies.

Items		Lifestyle modification + Herbal medicine	Lifestyle modification + Placebo
Participants			
Total number (Male/Female)		326 (166/160)	277 (137/140)
Mean number ± SD		40.8 ± 22.5	37.6 ± 20.8
Mean age of participants^a^		46.3 ± 6.4	46.7 ± 6.3
Study duration, week		12.0 ± 0.0	
Country (n of study)		Iran (4), China (3), South Korea (1)	
Intra-hepatic outcomes (n of study, participants)			
Liver steatosis using US (3, 193)	Grade 1, %	35.8 ± 22.4	38.6 ± 22.3
	Grade 2, %	49.7 ± 19.0	39.0 ± 25.0
	Grade 3, %	15.7 ± 4.7	5.8 ± 3.2
CT liver/spleen ratio (2, 166)^a^		0.79 ± 0.01	0.77 ± 0.05
Hepatic enzymes (U/L)^a^	ALT (8, 603)	56.6 ± 23.4	54.5 ± 21.0
	AST (8, 603)	38.3 ± 14.9	35.6 ± 12.4
	GGT (4, 603)	41.6 ± 19.5	45.3 ± 21.0
Extra-hepatic outcomes (n of study, participants)			
Triglyceride (mg/dL,7, 504)^a^		197.3 ± 44.7	220.2 ± 65.5
Total cholesterol (mg/dL, 7, 504)^a^		200.7 ± 19.6	195.5 ± 22.0
BMI (kg/m^2^, 7, 504)^a^		29.0 ± 2.2	28.6 ± 1.8
HOMA-IR (6, 480)^a^		3.9 ± 1.5	3.6 ± 0.4

US, ultrasound; CT, computed tomography; ALT, alanine transaminase; AST, aspartate transaminase; GGT, gamma-glutamyl transferase; BMI, body mass index; HOMA-IR, homeostatic model assessment for insulin resistance.

^a^ The mean was estimated using the presented mean value of each study.

Regarding LM, all participants in both groups were guided to restrict the high-carbohydrate and high-fat foods, and to increase their physical activity to at least 150 min per week. Herbal medicine group were administered with multi-herbal formulas [Yiqi Sanju Formula: 益气散聚方 (Lou et al., 2008), Tiaogan Lipi Recipe: 调肝理脾方 (Yu et al., 2015), Lingguizhugan Decoction: 苓桂术甘汤 (Dai et al., 2021)] or single-herb extract [*Glycyrrhiza glabra* L.: 甘草 (Rostamizadeh et al., 2022), *Magnoliae officinalis*: 厚朴 (Jeong et al., 2017), *Trigonella Foenum-graecum* L. semen: 胡芦巴 (Babaei et al., 2020), *Portulaca oleracea* L.: 马齿苋 (Darvish Damavandi et al., 2021), and *Rhus Coriaria* L. fructus (Kazemi et al., 2020)], respectively (Supplementary Table S1). After 12-week intervention, the changes of liver steatosis (US and/or CT), liver enzymes (ALT, AST, and GGT) and/or extra-hepatic parameters (BMI, HOMA-IR, TG and TC) were evaluated.

### 3.2 Change in hepatic steatosis

**FIGURE 2:**
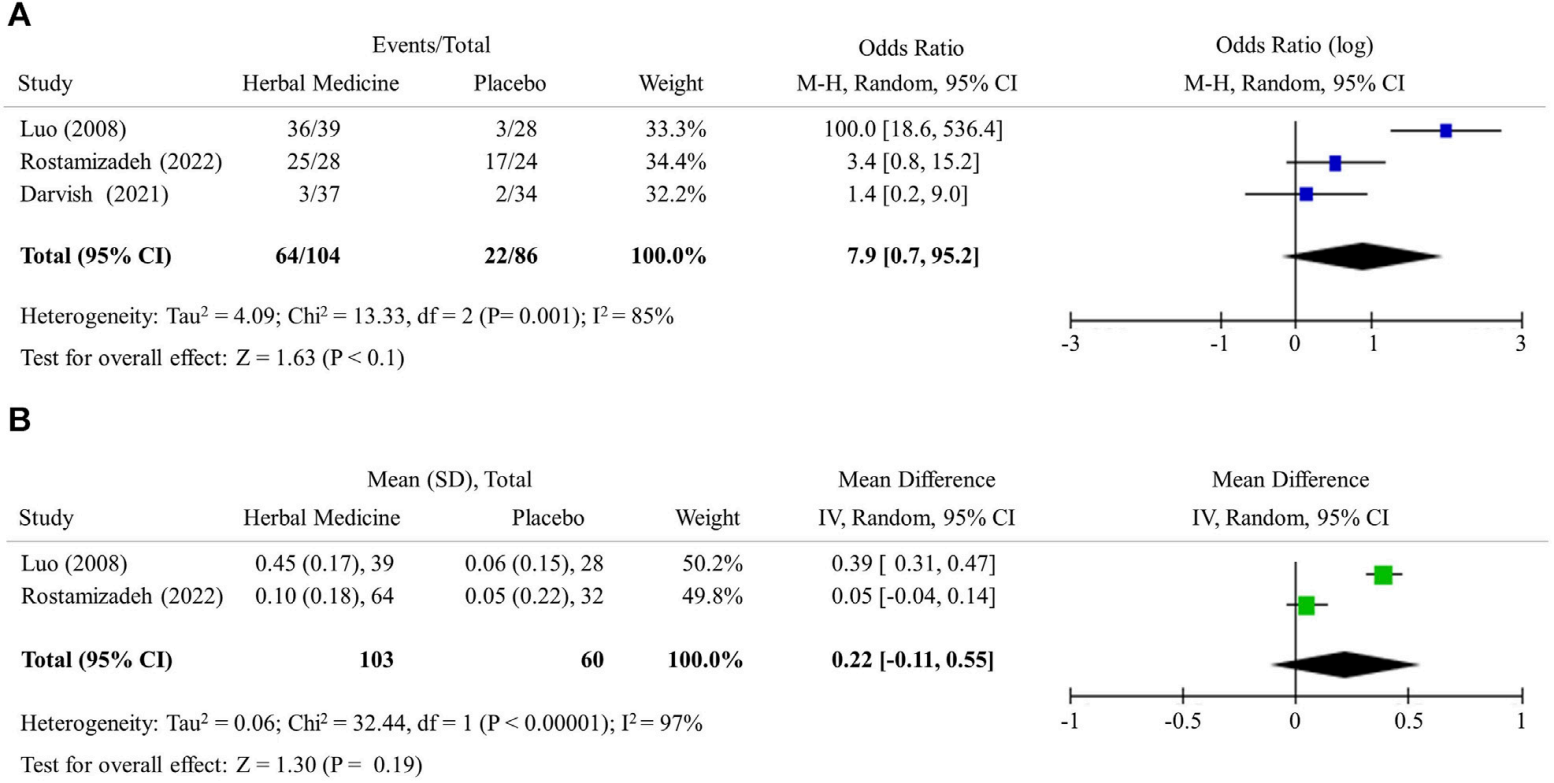
Hepatic steatosis. **(A)** Improvement in ultrasound liver steatosis grade **(B)** Computed tomography liver/spleen ratio.

The US-based measurement of liver steatosis (3 studies, 190 participants) revealed that herbal medicines notably increased the case of improvement in liver steatosis grade: as OR = 7.9 (95% CI 0.7 to 95.2, *p* < 0.1) (Figure 2A). Meanwhile, CT-based liver/spleen ratio (2 studies, 163 participants) did not show the significant mean difference between 2 groups as 0.2 (95% CI −0.1 to 0.6, *p* = 0.19) (Figure 2B).

### 3.3 Change in hepatic inflammation

**FIGURE 3:**
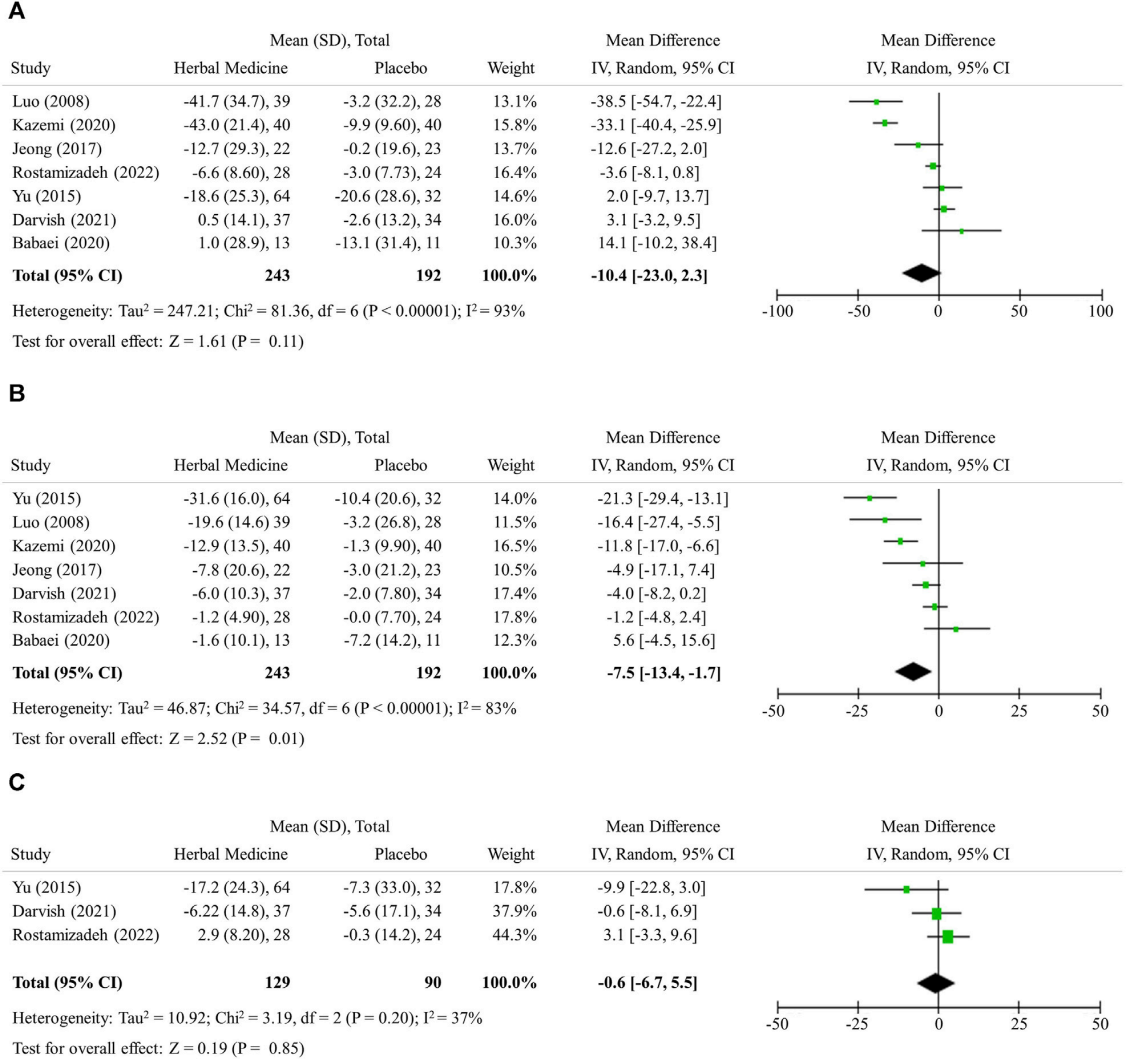
Hepatic inflammation. **(A)** Alannine transaminase **(B)** Aspartate transaminase **(C)** Gamma-glutamyl transferase.

The hepatic enzyme levels were further lowered by herbal medicines compared with LM alone, however no statistical significance was observed in the mean difference; ALT −10.34 (95% CI −23.0 to 2.3, 7 studies, 435 participants), AST −7.5 (95% CI −13.4 to −1.7, 7 studies, 435 participants), and GGT −0.6 (95% CI −6.7 to 5.5, 3 studies, 219 participants), respectively (Figures 3A–C).

### 3.4 Change in lipid profile

**FIGURE 4:**
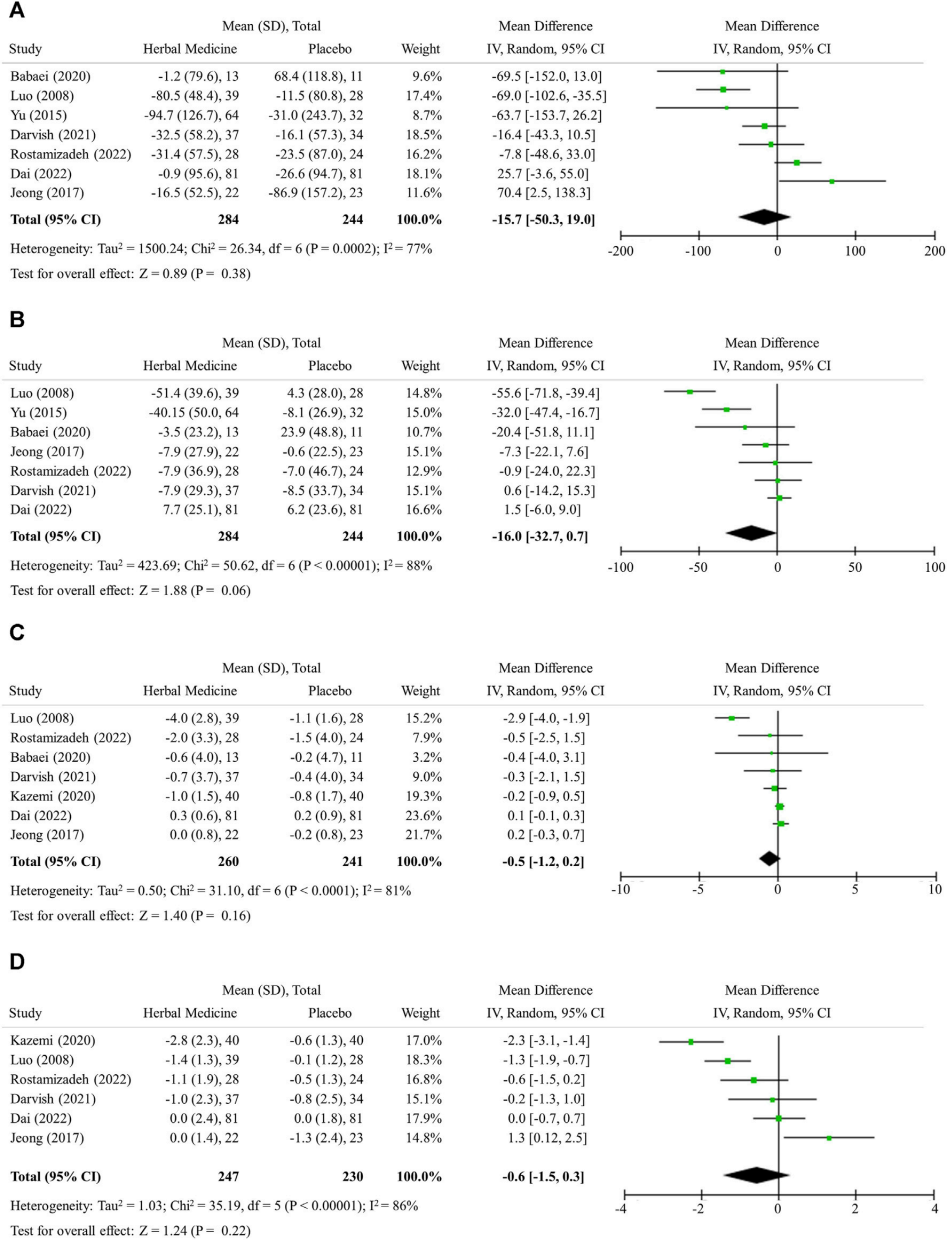
Lipid profile, obesity, and insulin resistance. **(A)** Triglyceride **(B)** Total cholesterol **(C)** Body mass index **(D)** HOMA-IR.

The herbal medicines further lowered serum levels of both TG and TC, but without statistical significance in mean difference, likely −15.7 (95% CI -50.3 to 19.0, 7 studies, 528 participants) and −16.0 (95% CI −32.7 to 0.7, 7 studies, 528 participants), respectively (Figures 4A, B).

### 3.5 Obesity and insulin resistance

The herbal medicines further lowered serum levels of both BMI and HOMA-IR, but without statistical significance in mean difference; −0.5 (95% CI −1.2 to 0.2, 7 studies, 501 participants) and −0.6 (95% CI −1.5 to 0.3, 6 studies, 477 participants), respectively (Figures 4C, D).

## 4 Discussion

NAFLD is attracting attention from the medical community due to its high prevalence worldwide and as a major components of metabolic syndrome as well as chronic liver diseases (Wong et al., 2023). LM is currently the only way to control this disease, which underscores the demand for the development of effective treatments (Younossi et al., 2021). Based on the partial evidence for the potential of herbal medicines against NAFLD (Dai et al., 2021). We herein performed a meta-analysis to evaluate the additive effect of herbal medicines on LM in the treatment of NAFLD.

Biopsy-derived histological examination is a classical standard to assess the levels of steatosis, inflammation and fibrosis in the liver. Biopsy is however invasive; thus, the multiple tests are almost impossible (Segura-Azuara et al., 2022). Accordingly, US and/or CT examinations are primary tools when evaluating intervention-induced changes in clinics and RCTs, (Schwenzer et al., 2009). As a non-invasive method, US detects fat accumulation in the liver by observing ultrasound attenuation and CT measures the density of the tissue passed by the X-ray beam and identifies fatty liver by comparing the density of liver with that of the spleen. If the ratio of the density of liver to that of spleen is less than 1.0, it is diagnosed with fatty liver. Transient elastography which is an enhanced form of ultrasound detect liver fibrosis in NAFLD or other liver diseases (Sanyal et al., 2023). In this study, 3 RCTs (Lou et al., 2008; Darvish Damavandi et al., 2021; Rostamizadeh et al., 2022) employed US examination and the meta-analysis showed the effect of herbal medicines in improving hepatic steatosis was 7.9 times higher than LM alone (Figure 2A). Two RCTs (Lou et al., 2008; Yu et al., 2015) utilized CT examination and it appeared that herbal medicines had the effect of increasing the liver to spleen ratio to closer to 1.0 in the meta-analysis (Table 1; Figure 2B).

In addition to histological examination, the levels of ALT, AST, and GGT are widely used to check for the damage of hepatic cells and inflammation in the liver. The reference ranges for ALT, AST, and GGT can vary depending on the laboratory and the method used for testing. However, on average, normal ranges are ALT: 0–45 IU/L, AST: 0–35 IU/L, GGT: 0–30 IU/L (Paul, 2020). If fatty liver exists but there is no relevant hepatic cell damage or inflammation, the ALT, AST, and GGT levels should be within normal range. In this study, herbal medicines were shown to have additive effect to LM in decreasing ALT and AST levels by about 10 (Figures 3A, B). This degree of the changes can be regarded as the extent to which herbal medicines normalize the ALT level, since the baseline ALT level of the subjects were around 55 (Table 1). However, it should be noted that the number of studies included in the analysis is small, which limits the interpretation of the results in terms of selection bias and statistical significance.

It has been known that NAFLD is present in up to 75% of overweight people and in more than 90% of people with severe obesity (Rinella et al., 2023). Obesity significantly contributes to NAFLD progression by disrupting lipid metabolism and promoting systemic inflammation. This dysregulation exacerbates hepatic fat accumulation, advancing NAFLD. Additionally, obesity-induced inflammation extends beyond the liver, impacting systemic inflammation and worsening underlying NAFLD processes through increased oxidative stress, mitochondrial dysfunction, and gut dysbiosis (Lim et al., 2021). Therefore, patients with NAFLD are recommended to lose weight through LM. Recently, GLP-1R agonists have been shown that they effectively reduce body weight and may aid in reversing NAFLD (Andreasen et al., 2023). Herbal medicines have been extensively studied that they help reduce body weight and attenuate NAFLD by suppressing appetite and reducing oxidative stress, improving mitochondrial function, and modulating intestinal dysbiosis (Dai et al., 2021).

In this study, the additive effect of herbal medicines in reducing BMI was not found to be significant (Figure 4C). It has been known that weight loss more than 5% (BMI reduction of 1.5 or more, assuming a patient with BMI 30) can improve NAFLD (Younossi et al., 2021). However, LM alone (placebo) did not sufficiently reduce BMI in the studies included in this analysis (Figure 4C). Considering that many patients find it difficult to successfully lose weight through LM, herbal medicines might help weight loss when combined with LM. When HOMA-IR drops below 2, it is regarded that insulin resistance is improved (Isokuortti et al., 2017). In situation where LM alone does not improve HOMA-IR enough, herbal medicine treatment can help to some extent (Figure 4D). In addition, although the levels of TG and TC were in the normal range at baseline, they were reduced by herbal medicines confirming the effect of improving lipid metabolism as previously known (Dai et al., 2021). However, further study on herbal medicines is needed to ensure that herbal medicines can effectively treat metabolic disorders as well as NAFLD.

The herbal medicines and their doses used in the RCTs included in this study are listed in Supplementary Table S1. There was not significant safety issue in the RCTs. Regarding the efficacy of the herbal medicines used in the RCTs included in this study, it has been extensively studied for anti-oxidant, anti-inflammatory, hypoglycemic, and lipid-lowering effects of *Astragali* Radix, *Atractylodis* Rhizoma Alba, *Salviae Miltiorrhizae* Radix, *Bupleuri* Radix, *Artemisiae* Capillaris Herba, *Polygoni Cuspidati* Radix, *Cassiae* Semen, *Crataegi* Fructus, *Poria Sclerotium*, *Cinnamomi Ramulus*, *Glycyrrhiza* Rhizoma in the treatment of NAFLD (Dai et al., 2021). In addition, several studies demonstrated *Coptidis* Rhizoma (Li et al., 2024), *Polygoni orientalis* (Chen et al., 2021), *Magnoliae offcinalis* (Kuo et al., 2020), *and Tegillarca granosa* L. (Jiang et al., 2024) improve glucose and lipid metabolism by modulating PI3K-AKT and AMPK signaling pathway, and *Coicis* Semen (Chiang et al., 2020), *Cyperi* Rhizoma (Wang et al., 2022), *Verbena officinalis* L. (Kubica et al., 2020), *Trigonella Foenum-graecum* L. semen (Yadav and Baquer, 2014), *Portulaca oleracea* L. (Rahimi et al., 2019), and Rhus Coriaria L. fructus (Alsamri et al., 2021) exhibit anti-oxidant, anti-inflammatory effects by enhancing superoxide dismutase and glutathione activity and inhibiting the production of inflammatory mediators such as nitric oxide, tumor necrosis factor-α, interleukin-1β, interleukin-6, and prostaglandin E2. Since the ingredients and their chemical structure of herbal medicines above have been identified (Abu-Reida et al., 2014; Zhou et al., 2015; Pang et al., 2016; Dong et al., 2017; Yang et al., 2017; Wang et al., 2018; Luo et al., 2019; Wang et al., 2019; Kubica et al., 2020; Li et al., 2020; Liu et al., 2020; Zhu et al., 2020; Chen et al., 2021; Hsueh et al., 2021; Xue et al., 2021; Zhang et al., 2021; Chang et al., 2022; Lu et al., 2022; Singh et al., 2022), it is expected that therapeutics will be developed through analog development based on the previous studies.

The limitations of this study are as follows. First, although total 30 RCTs were conducted using herbal medicines for the treatment of NAFLD (Figure 1), there were a few studies using placebo control, so a sufficient number of studies could not be analyzed. Second, the levels of AST, GGT, TG, and TC at baseline were within normal range, which suggest that the patients included in this analysis had mild NAFLD. Third, there were not many studies in which imaging were conducted, even in studies mainly targeting patients with mild NAFLD. In addition, it is imperative to address the role of genetic and epigenetic factors in NAFLD. The examination of genetic (e.g., *PNPLA3*, *TM6SF2*, *MBOAT7*, and *TMC4* variants) and epigenetic (e.g., DNA methylation) factors becomes crucial in understanding the multifaceted nature of NAFLD progression (Younossi et al., 2018). Several studies have shown that *PNPLA3* genotype and characteristic epigenetic alterations vary depending on nationality and ethnicity (Szanto et al., 2019; Krawczyk et al., 2020). Therefore, it is necessary to consider genetic and epigenetic factors depending on nationality and ethnicity when applying the finding of this study, given that the RCTs included in this study predominantly originate from Asian and Middle Eastern countries (China, South Korea, and Iran).

## 5 Conclusion

Given some limitations above, this systematic review and meta-analysis at least partially evidenced the add-on efficacy of herbal medicines on LM in the treatment of NAFLD, which produce a reference data for herb-derived drug developments against NAFLD in the future. The further well-designed and larger scaled RCTs are however necessary to provide a solid basis for NAFLD treatment.

## Data Availability

The original contributions presented in the study are included in the article/Supplementary Material, further inquiries can be directed to the corresponding authors.
