# Supplementary figures and images for: The additive effect of herbal medicines on lifestyle modification in the treatment of non-alcoholic fatty liver disease: a systematic review and meta-analysis
Source: Front Pharmacol. 2024 Feb 23;15:1362391. doi: 10.3389/fphar.2024.1362391 (PMC10920213; doi:10.3389/fphar.2024.1362391)

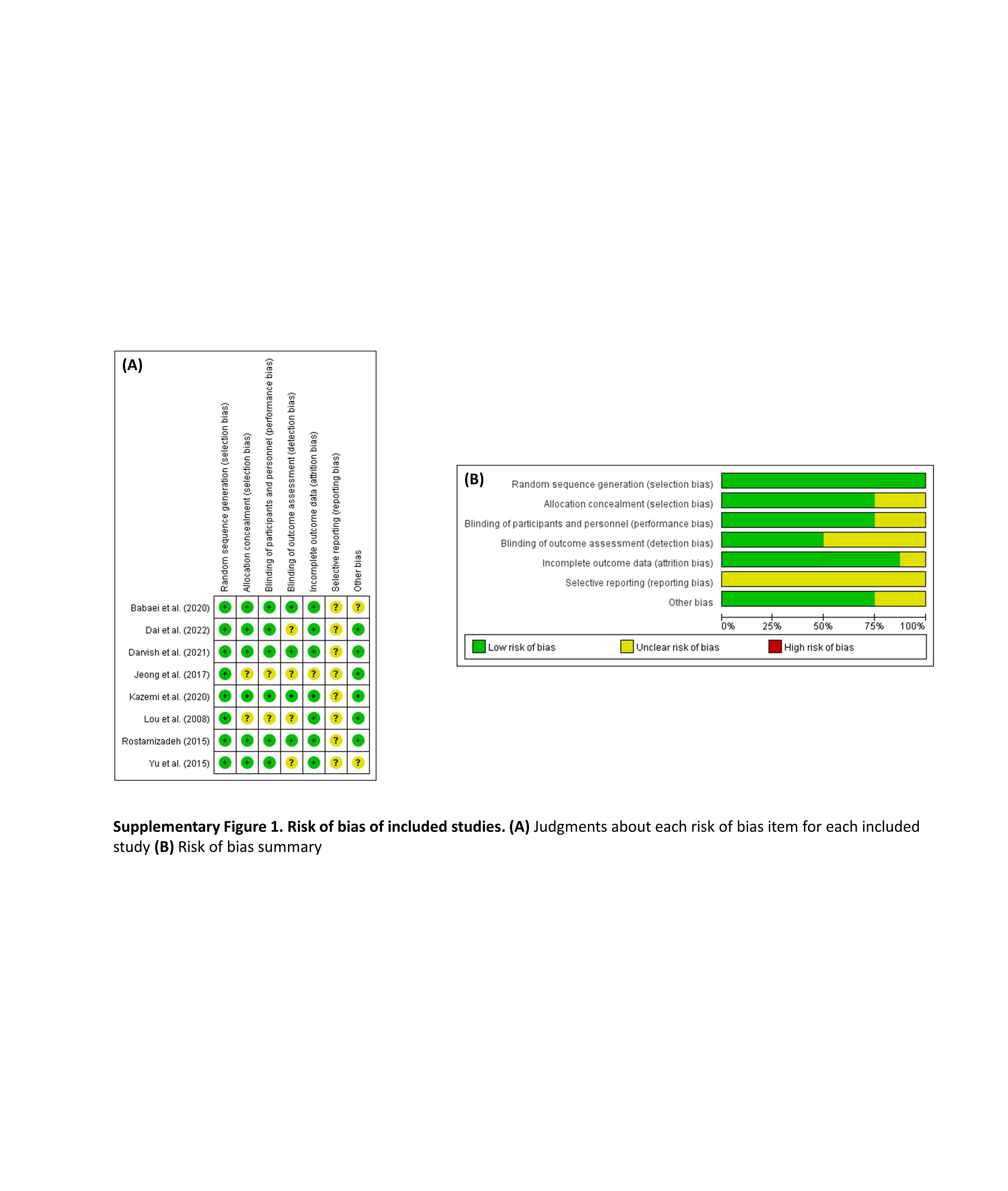

Supplement: Supplementary file 1 [file Image1.JPEG]
