# Supplementary material for: The additive effect of herbal medicines on lifestyle modification in the treatment of non-alcoholic fatty liver disease: a systematic review and meta-analysis
Source: Front Pharmacol. 2024 Feb 23;15:1362391. doi: 10.3389/fphar.2024.1362391 (PMC10920213; doi:10.3389/fphar.2024.1362391)
Supplement: Supplementary file 2 [file Table2.DOCX]

| **Supplementary Table 1. Detailed information of included studies** | | | | | | | | | | | | |
| --- | --- | --- | --- | --- | --- | --- | --- | --- | --- | --- | --- | --- |
| First author | Participants (M/F) | Herbal medicine | Outcomes | | | | | | | | | |
| (year) | HM:Placebo | (Chinese, dose per day) | Primary | | | | | Secondary | | | | |
|  |  |  | US | CT | ALT | AST | GGT | BMI | TG | TC | HOMA-IR | |
| Lou et al.  (2008) [1] | 67 (44/23)  39:28 | Yiqi Sanju Formula  (益气散聚方, extract granules^1^, 2 times) | O | O | O | O |  | O | O | O | | O |
| Yu et al.  (2015) [2] | 99 (52/47)  66:33 | Tiaogan Lipi Recipe  (调肝理脾方, extract granules^2^, once) |  | O | O | O | O |  | O | O | |  |
| Dai et al.  (2022) [3] | 162 (68/94)  81:81 | Lingguizhugan Decoction  (苓桂术甘汤, extract granules^3^, 2 times) |  |  | O | O | O | O | O | O | | O |
| Rostamizadeh  (2015) [4] | 52 (0/52)  28:24 | *Glycyrrhiza glabra* L. extract^4^  (甘草, 500mg, 2 times) | O |  | O | O | O | O | O | O | | O |
| Jeong et al.  (2017) [5] | 45 (40/5)  22:23 | *Magnoliae officinalis* extract  (厚朴, 400mg, once) |  |  | O | O |  | O | O | O | | O |
| Babaei et al.  (2020) [6] | 24 (22/2)  13:11 | *Trigonella Foenum-graecum* L. semen extract  (胡芦巴, 500mg, 2 times) |  |  | O | O |  | O | O | O | |  |
| Darvish et al.  (2021) [7] | 74 (43/31)  37:37 | *Portulaca oleracea* L. extract  (马齿苋, 300mg, once) | O |  | O | O | O | O | O | O | | O |
| Kazemi et al.  (2020) [8] | 80 (34/46)  40:40 | *Rhus Coriaria* L. fructus extract  ( * , 500mg, 4 times) |  |  | O | O |  | O |  |  | | O |
| ^1^ Extract granules mainly composed of *Astragali* Radix (黄芪) and *Coptidis* Rhizoma (黄连)  ^2^ Granules extracted from *Astragali* Radix (黄芪) 20 g, *Atractylodis* Rhizoma Alba (白朮) 15 g, *Coicis* Semen (薏苡仁) 20 g, *Salviae Miltiorrhizae* Radix (丹参) 20 g, *Cyperi* Rhizoma (香附) 10 g, *Bupleuri* Radix (柴胡) 10 g, *Artemisiae* Capillaris Herba (茵陈) 10 g, *Polygoni Cuspidati* Radix (虎杖) 10 g, *Tegillarca granosa* L. (瓦楞子) 20 g, *Verbena officinalis* L. (马鞭草) 15 g, *Cassiae* Semen (决明子) 15 g, *Crataegi* Fructus (山楂) 15 g, and *Polygoni orientalis* fructus (水红花子) 15 g  ^3^ Granules extracted from *Poria Sclerotium* (茯笭) 12 g, *Cinnamomi Ramulus* (桂枝) 9 g, *Atractylodis* Rhizoma Alba (白朮) 6 g, and *Glycyrrhiza* Rhizoma (甘草) 6 g  ^4^ Containing 24 % monoammonium glycyrrhizinate  ^*^ In traditional Chinese Medicine and traditional Korean Medicine, the resin of *Rhus verniciflua* Stokes is used as 乾漆 | | | | | | | | | | | | |

1. Lou, S.Y., et al., *[Effects of Yiqi Sanju Formula on non-alcoholic fatty liver disease: a randomized controlled trial].* Zhong Xi Yi Jie He Xue Bao, 2008. **6**(8): p. 793-8.

2. Yu, Q., et al., *[Treating non-alcoholic fatty liver disease patients of Gan stagnation Pi deficiency syndrome by tiaogan lidi recipe: a randomized controlled clinical trial].* Zhongguo Zhong Xi Yi Jie He Za Zhi, 2015. **35**(4): p. 401-5.

3. Dai, L., et al., *Lingguizhugan Decoction, a Chinese herbal formula, improves insulin resistance in overweight/obese subjects with non-alcoholic fatty liver disease: a translational approach.* Front Med, 2022. **16**(5): p. 745-759.

4. Rostamizadeh, P., et al., *Effects of licorice root supplementation on liver enzymes, hepatic steatosis, metabolic and oxidative stress parameters in women with nonalcoholic fatty liver disease: A randomized double-blind clinical trial.* Phytother Res, 2022. **36**(10): p. 3949-3956.

5. Jeong, J.Y., et al., *New botanical drug, HL tablet, reduces hepatic fat as measured by magnetic resonance spectroscopy in patients with nonalcoholic fatty liver disease: A placebo-controlled, randomized, phase II trial.* World J Gastroenterol, 2017. **23**(32): p. 5977-5985.

6. Babaei, A., et al., *Comparison of the efficacy of oral fenugreek seeds hydroalcoholic extract versus placebo in nonalcoholic fatty liver disease; a randomized, triple-blind controlled pilot clinical trial.* Indian J Pharmacol, 2020. **52**(2): p. 86-93.

7. Darvish Damavandi, R., et al., *Effect of Portulaca Oleracea (purslane) extract on liver enzymes, lipid profile, and glycemic status in nonalcoholic fatty liver disease: A randomized, double-blind clinical trial.* Phytother Res, 2021. **35**(6): p. 3145-3156.

8. Kazemi, S., et al., *The effects of sumac (Rhus coriaria L.) powder supplementation in patients with non-alcoholic fatty liver disease: A randomized controlled trial.* Complement Ther Clin Pract, 2020. **41**: p. 101259.
